# The Effect of Lysolecithin Supplementation on Growth Performance, Nutrient Digestibility, Intestinal Morphology, and Lipid Metabolism in Yellow-Feathered Broilers Fed Diets with Different Fat Levels

**DOI:** 10.3390/ani15182752

**Published:** 2025-09-20

**Authors:** Ying Zhang, Leilei Zhu, Zheng Luo, Jie Feng

**Affiliations:** 1 Kemin (China) Technologies Co., Ltd., Zhuhai 519040, China; johnny.zhang@kemin.com (Y.Z.); leo.luo@kemin.com (Z.L.); 2 Key Laboratory of Animal Nutrition and Feed Sciences of Zhejiang Province, College of Animal Science, Zhejiang University, Hangzhou 310058, China; zhuleilei573122@163.com

**Keywords:** lysolecithin, yellow-feathered broiler, growth performance, nutrient digestibility, lipid metabolism

## Abstract

This study was conducted to investigate the interaction of dietary fat level and lysolecithin (LL) supplementation on the growth performance, nutrient digestibility, intestinal morphology, intestinal lipase activity, and lipid metabolism in yellow-feathered broilers (YFBs) during day 1–50. A total of 384 one-day-old male broilers were assigned to a 2 × 2 factorial design, including dietary fat level (Normal-fat, NF: 3% lard, day 1–21; 4.5% lard, day 22–50/Low-fat, LF: 2.4% lard, day 1–21; 3.6% lard, day 22–50) and LL supplementation (0% or 0.05%). Each treatment had six replicates with 16 birds each. The trial lasted 50 days from day 1 to day 50. During the starter phase (day 1–21), birds fed the NF diet exhibited significantly greater average daily gain (ADG) and a lower feed conversion ratio (FCR) compared to those fed the LF diet (*p* < 0.05). Supplementation with LL significantly improved ADG and reduced FCR throughout all phases (day 0–21, day 22–50, and day 0–50) (*p* < 0.05). At day 21, LL also increased the apparent metabolizable energy (AME) and the digestibility of crude protein and ether extract (*p* < 0.05). LL supplementation increased the villus height-to-crypt depth (VH:CD) ratio in the small intestine and elevated intestinal lipase activity during the experimental period (*p* < 0.05). Regarding lipid metabolism, LL supplementation decreased serum total cholesterol (TC) and the LDL:HDL ratio, while increasing liver total lipase (TL) activity at both day 21 and day 50 (*p* < 0.05). These findings suggest that dietary supplementation with 0.05% LL improves growth performance, enhances nutrient digestibility and intestinal absorptive capacity, and positively modulates lipid metabolism in YFBs.

## Simple Summary

As bio-emulsifier, Lysolecithin (LL) is widely used in broiler diets “on top” to achieve better growth performance or reformulated diets where lipids were reduced to maintain bird performance at lower cost. However, little information is available regarding its use in yellow-feathered broilers (YFBs). The present study aimed to explore the effects of dietary fat level and LL supplementation on growth performance, nutrient digestibility, intestinal morphology, lipase activity, and lipid metabolism in YFBs. The results showed that LL supplementation improved nutrient digestion and absorption, enhanced energy utilization, and positively affected intestinal morphology and lipid metabolism, suggesting that LL is a practical nutritional strategy for YFB production.

## 1. Introduction

The yellow-feathered broiler (YFB), one of China’s three primary broiler types alongside standard white broilers and hybrid strains derived from broiler-layer crosses [1], holds significant economic and cultural importance. Annual production exceeds 4.9 billion birds and continues to grow steadily [2,3], reflecting its prominent role in China’s poultry sector. Based on fattening duration, YFBs are categorized into three subtypes: fast-growing (49–56 days), medium-growing (70–90 days), and slow-growing (120–180 days). Compared to commercial white broilers (e.g., Cobb and Arbor Acres), YFBs exhibit markedly slower growth rates [1], a trait intrinsically linked to breeding strategies prioritizing distinctive meat quality, flavor, and texture. This intentional selection has resulted in genetically constrained nutrient utilization efficiency [4].

Lipids are extensively incorporated into poultry diets to enhance dietary metabolizable energy (ME) density, improve the bioavailability of fat-soluble vitamins (A, D, E, K), optimize feed palatability, reduce dust formation, and augment pellet durability during feed processing [5,6,7]. In addition, dietary lipids also facilitate the absorption of other fat-soluble nutrients (e.g., carotenoids) by promoting micelle formation in the intestine [8]. However, digestibility of lipids remains suboptimal in immature birds, primarily due to inadequate lipase activity and limited bile salt secretion during early post-hatch development [9]. For instance, fat digestibility in chicks aged <7 days can be ≤60% for saturated fats (e.g., beef tallow), rising to >80% by 3 weeks as endogenous enzyme systems mature. This physiological constraint is exacerbated by the high cost of lipid inclusion (often 5–9% of diet), which constitutes a major expense in broiler feed formulations [10]. Consequently, improving lipid digestibility is critical not only to reducing feed costs through optimized inclusion rates but also to maintain broiler growth performance without compromising carcass quality.

Lysolecithin (LL), an enzymatic hydrolysis product of lecithin derived from soybean oil extraction [11] exhibits unique physicochemical properties critical to its biological functions. Structural modification via fatty acid removal confers LL with superior hydrophilicity and oil-in-water emulsification capacity compared to intact lecithin [12]. Studies reported that dietary supplementation of LL enhanced apparent metabolizable energy (AME) utilization, improved lipid digestibility coefficients, and increased retention of fat-soluble nutrients in broilers. On one hand, LL reduces interfacial tension between lipid droplets and intestinal fluids, facilitating micelle formation and increasing fat accessibility to lipases [13]. On the other hand, LL integrates into phospholipid bilayers of enterocytes, increasing membrane fluidity and permeability. This promotes nutrient transporter activity and ion channel function, thereby enhancing absorption efficiency. Concurrently, LL-mediated membrane remodeling enhances microvillus structural integrity, as evidenced by increased villi height-to-crypt depth ratios (VH:CD) and collagen matrix maturation [14], upregulating collagen-coding genes (e.g., *COL1A1*, *COL3A1*), strengthening villus collagen cross-linkages, and supporting intestinal morphology [15]. Additionally, supplementation of LL could attenuate pathological lipid accumulation by enhancing mitochondrial β-oxidation and very-low-density lipoprotein (VLDL) assembly, thereby reducing hepatocellular steatosis risk [16].

Commercial diets for yellow-feathered chickens (YFCs) in China typically incorporate 3–8% added fats or oils to meet energy demands [3]. However, YFCs exhibit intrinsic physiological distinctions from modern, white-feathered broilers (e.g., Arbor Acres), including slower growth rates and reduced nutrient digestibility [17,18]. However, existing studies predominantly focus on the effects of LL on growth performance of fast-growing broilers rather than slow-growing YFBs. Furthermore, the insights into lipid metabolism of LL supplementation were lacking in broilers fed diets with different fat levels. Therefore, the aim of this study was to investigate the effect of LL supplementation on growth performance, nutrient digestibility, intestinal morphology, and lipid metabolism in YFBs fed diets with different fat levels.

## 2. Materials and Methods

### 2.1. Experimental Design and Diet

The trial was conducted at a commercial poultry facility (Zhejiang Qun Da Livestock Farming Co., Ltd., Haining, China). The animal care protocol for this study was approved by the Animal Care Committee of Zhejiang University (No. ZJU20220501).

A total of 384 one-day-old male YFB chicks (initial body wight, BW: 39.78 ± 0.16 g; coefficient of variation, CV = 4.02%) were selected and then were randomly assigned to four dietary treatments in a 2 × 2 factorial, including dietary fat level (Normal-fat, NF: 3% lard, day 1–21; 4.5% lard, day 22–50/Low-fat, LF: 2.4% lard, day 1–21; 3.6% lard, day 22–50) and LL supplementation (0% or 0.05%). The LL complex used in this study was provided by Kemin (China) Technologies Co., Ltd. (Zhuhai, China). Each treatment included six replicate cages and 16 birds per cage. The basal diet was corn–soybean meal (SBM)-based, formulated to meet NRC (1994) [19] nutrient requirements for broilers. Diets were phased into two stages: starter (day 1–21) and grower (day 22–50) (Table 1).

### 2.2. Housing and Management

Birds were housed in stainless-steel battery cages (bi-layer design; 16 birds/cage) within a temperature-controlled room (relative humidity: 60–70%). Ad libitum access to feed and water was provided via nipple drinkers and trough feeders. The trial lasted for 50 days and was divided into the starter period (day 1–21) and the grower period (day 22–50). The photoperiod was kept at 24 hours L: 0 hours D (day 1–7) and transitioned to 23 hours L: 1 hours D (day 8–21). Temperature settings were adjusted to 32–35 °C (day 1–7), reduced to 30–32 °C (day 8–14), then decreased by 2 °C weekly until reaching 24–26 °C on day 21. The vaccination protocol of yellow-feathered chicks was conducted following the guidelines of the commercial poultry facility (Zhejiang Qun Da Livestock Farming Co., Ltd., Haining, China).

### 2.3. Growth Parameters and Apparent Nutrient Digestibility

Feed intake per replicate was recorded daily. Average daily gain (ADG), average daily feed intake (ADFI), and feed conversion ratio (FCR) were measured on days 0, 21, and 50, and overall performance traits were calculated for the entire period from day 1 to 50 of age.

On day 19 to 21 and day 47 to 49 of the study, excreta samples were collected, frozen, and freeze-dried for later analysis. Feed intake and total excreta were collected twice daily during the 3-day balance trial. The excreta were immediately mixed with 10 mL of 10% HCl per 100 g and pooled by cage and stored at −20 °C until analysis. Before calorie determination, the excreta were dried in a forced air oven at 65 °C for 72 h and ground to pass a 0.425 mm screen. The dry matter (DM), crude protein (CP), ether extract (EE), and crude fiber (CF) of excreta and feed samples were analyzed according to the described protocol by AOAC (2012) [21]. The gross energy (GE) of excreta and feed samples were determined by using a Parr 6100 oxygen bomb calorimeter (Parr Instrument Co., Moline, IL, USA) to measure their heat of combustion. Apparent metabolizable energy (AME) was calculated as (GE intake − GE excreta) ÷ feed intake.

### 2.4. Blood, Digesta and Organs Sampling, and Intestinal Morphology

On day 21 and 50 of the study, one bird per replicate with similar body weight was selected and euthanized using carbon dioxide for the collection of blood and tract samples. About 10 mL of blood sample was collected from each bird. The serum samples were obtained after the blood was centrifuged at 3000 rpm for 10 min and stored at −20 °C until analysis. The duodenum, jejunum, and ileum samples were then placed in sealed bags and stored at −20 °C. The duodenal, jejunal, and ileal digesta, as well as liver and pancreas samples, were immediately collected and stored at –80 °C to preserve enzymatic activity, metabolites, and other biochemical components until subsequent analysis.

Serum glucose (GLU), total cholesterol (TC), triglycerides (TG), high-density lipoprotein (HDL), and low-density lipoprotein (LDL) levels were measured using the Accutrend^®^ Plus system portable analyzers (F. Hoffmann-La Roche AG, Basel, Switzerland). The concentration of serum free fatty acid (FFA) was analyzed by gas chromatography using a capillary column (AT-FFAP: 30 m × 0.32 mm × 0.5 μm).

The segments of duodenum, jejunum, and ileum were fixed in 4% paraformaldehyde and then embedded in paraffin wax for intestinal morphology study. Two sections of 5 μm were cut from each piece and stained with hematoxylin and eosin. VH and CD were measured in intestinal cross-sections with four well-oriented crypt-villus units. A total of eight units were collected for each bird using an image processing and analysis system (Leica Imaging Systems, Cambridge, UK), and the values were averaged on a per bird basis.

An amount of 1.2 g of each sample from the liver, pancreas, and digesta (collected from the duodenum, jejunum, and ileum, respectively) were weighed and allowed to thaw at room temperature. Then, they were homogenized in normal saline with a concentration of 10% *w*/*v*. After centrifugation at 2500 r/m for 10 min, the supernatants from the liver were analyzed for hepatic lipase (HL), lipoprotein lipase (LPL), and total lipase (TL). Similarly, the supernatants from the digesta were analyzed for pancreatic lipase (PL). All enzymatic determinations were conducted using ELISA kits (Nanjing Jiancheng Bioengineering Institute, Nanjing, China).

### 2.5. Statistical Analysis

All data were analyzed by 2-way ANOVA using the general linear model procedure of SAS 9.2 (SAS Institute, Cary, NC, USA, 2010). The model included the main effects of dietary LL, dietary fat, and their interactions. Either one replicate served as an experimental unit. Differences among means were tested by the LSD method, and statistical significance was set at *p* < 0.05.

## 3. Results

### 3.1. Growth Performance and Nutrient Utilization

No significant interaction (*p* > 0.05) between dietary fat level (NF vs. LF) and LL supplementation on growth parameters (*p* > 0.05) was observed (Table 2). Broilers fed NF diets exhibited greater ADG (+8.7%, *p* < 0.05) and ADFI (+4.0%, *p* < 0.05) with reduced FCR (−4.6%, *p* < 0.05) compared to LF groups during day 1–21. LL supplementation increased ADG (+1.8–16.4%, *p* < 0.05) and reduced FCR (−1.4–8.1%, *p* < 0.05) compared with non-supplemented controls during day 1–21, 22–50, and 1–50.

No interactions (*p* > 0.05) were detected between fat level and LL for AME or macronutrient digestibility (Table 3). NF diets increased AME by approximately 1.4–1.5% (*p* < 0.05) during both measurement periods (day 19–21 and 47–49). LL supplementation significantly enhanced AME (+0.9–2.5%), CP digestibility (+10.7–11.6%), and EE digestibility (+4.8–5.4%) on day 19–21 (*p* < 0.05), whereas no significant effects were observed on day 47–49.

### 3.2. Lipase and Liver Enzymes Activities

No interactions (*p* > 0.05) occurred between fat level and LL for digestive enzyme activities both on day 21 and day 50 (Table 4). LL supplementation showed 14–73% greater lipase activity (*p* < 0.05) of duodenum, jejunum, and pancreas (*p* < 0.05) at both sampling points. LL increased lipase activity in ileum by 13–30% at day 50 (*p* < 0.05) but not at day 21.

No interaction (*p* > 0.05) was observed between fat level and LL in the concentration of LPL, HL, and TL in the liver on day 21 (Table 5). There was significant interaction for TL, with the greatest value in the LF + LL group, and the lowest value in the LF + 0 LL group (interaction, *p* = 0.042) on d 50. LL supplementation significantly increased liver LPL, HL, and TL concentrations on day 21 and day 50 (*p* < 0.05).

### 3.3. Blood Biochemical Analyses

Serum concentration of HDL, LDL, LDL/HDL, and TC were not affected (*p* > 0.05) by dietary fat level and the interaction between fat level and LL on d 21 and 50 (Table 6). On day 21, the serum concentration of GLU was greatest (*p* < 0.05) in the NF + LL group and lowest in the NF +0 LL group, whereas the TG and FFA were lowest in the NF + LL group and greatest in the NF + LL group, respectively (interaction, *p* < 0.05). No difference (*p* > 0.05) was observed in serum concentrations of GLU, HDL, LDL, LDL, HDL, TC, TG, and FAA both on day 21 and day 50. The serum concentration of TC was less (*p* < 0.05) in the +LL group than in the +0 LL group.

### 3.4. Intestinal Morphology

The VH, CD, and VH:CD of duodenum, jejunum and ileum were not affected (*p* > 0.05) by the interaction between fat level and LL both on day 21 and 50 (Table 7). The VH, CD, VH:CD of duodenum, jejunum and ileum were not affected (*p* > 0.05) by dietary fat level except for VH:CD of duodenum on day 21. The VH and VH:CD of duodenum and ileum were greater (*p* < 0.05) in the +LL group than in the +0 LL group. The CD of jejunum and ileum were greater (*p* < 0.05) in the +LL group than in the +0 LL group on day 21, rather than day 50.

## 4. Discussion

The observed growth-promoting effects of dietary fat supplementation align with established principles of poultry nutrition. Consistent with Maiorka et al. [22], Niu et al. [23], and Ge et al. [24], our results demonstrate that increasing dietary energy density through fat inclusion (3–4.5% lard) improves feed conversion efficiency during the critical starter phase (day 1–21). This physiological response reflects the metabolic priority of lipid utilization in avian species, where exogenous fats provide concentrated energy substrates while reducing dietary heat increment compared to carbohydrates [25]. However, our findings reinforce the concept of an optimal inclusion threshold, as excessive fat levels may compromise digestive efficiency due to immature lipase secretion and bile salt limitations in young broilers [9,26]. No significant interaction between dietary fat level and LL supplementation was detected for most parameters (*p* > 0.05), suggesting that the effects of LL were independent of dietary fat content. These findings align with recent studies demonstrating that dietary emulsifiers can enhance energy utilization and growth performance in birds receiving both normal- and low-energy diets [27,28].

As reported by Zaefarian et al. [29], Allahyari-bake et al. [30], and Zampiga et al. [31], the beneficial effects of LL supplementation on growth performance observed in this study in yellow-feathered chickens exhibit a greater ADG and FCR across all growth phases. These improvements are likely due to the increased dual emulsification properties and membrane-modulating activities through LL addition, which resulted in enhanced lipid digestion and intestinal nutrient absorption [13]. Notably, our results revealed that 500 g/t LL supplementation effectively compensated for the reduced growth performance of broilers fed low-energy diets, corroborating Wealleans et al. [32], who reported that 57.88–73.11 kcal/kg ME could be recovered by the increased fat utilization efficiency of LL addition.

As animal-derived fat, lard contains 40–50% saturated fatty acids (SFA) compared to 12–18% in vegetable oils, creating a physical matrix less accessible to pancreatic lipase [33,34]. This structural limitation reduces lipolytic efficiency, particularly in young birds with immature bile salt secretion and lipase activity [35]. Our results demonstrate that LL supplementation significantly improved AM, crude protein (CP) digestibility, and ether extract (EE) digestibility at 21 days of age. These findings align with Jansen et al. [10], who reported LL-mediated improvements in DM digestibility (+6.3%) in lard-based broiler diets through enhanced micellar solubilization of saturated lipids. Similar effects have been reported in more recent studies using deactivated full-fat soybean and mixed fat sources, suggesting that the benefits of LL are generalizable across lipid types [28,36,37]. The surfactant properties of LL likely overcome the physical barriers imposed by SFA-rich substrates, as evidenced by 14–73% increases in intestinal lipase activity and improvements in villus morphology. Fats with low unsaturated-to-saturated (U:S) ratios (e.g., tallow, palm oil) typically impair crude fiber (CF) digestibility due to increased intestinal viscosity [35], which does not agree with our results. In this study, the experimental diets were corn–soybean meal–based, which are known to have low CF levels; this low CF content may explain why the expected effect of fat type on CF digestibility was not observed.

Intestinal lipase activity serves as a critical biomarker for dietary lipid utilization efficiency, reflecting the dynamic interplay between dietary fat composition, digestive enzyme secretion, and emulsification processes [38]. LLs, as amphiphilic biosurfactants, possess dual hydrophobic–hydrophilic domains that reduce lipid–water interfacial tension, facilitating micelle formation essential for pancreatic lipase action. This structural functionality enables LL to partition triacylglycerols into soluble aggregates, enhancing lipolytic cleavage onto absorbable monoacylglycerols and free fatty acids. Consistent with Lai et al.’s [38] findings on bile acid-mediated lipase activation, our results demonstrated that LL addition increases in duodenal and ileal lipase activities at day 21 and 50. The observed 14–73% increases in intestinal lipase activities align with the role of LL in stabilizing lipid-water interfaces, thereby maintaining optimal substrate orientation for catalytic cleavage [13], which corroborate ether extract and crude protein digestibility.

The LPL and hepatic lipase (HL) play pivotal roles in lipid homeostasis by hydrolyzing circulating chylomicron triglycerides (TG) and supplying non-esterified fatty acids (NEFA) for peripheral tissue utilization [39]. These enzymes, along with hormone-sensitive lipase (HSL), regulate systemic energy partitioning through coordinated TG hydrolysis and glycerol release [40]. Serum lipid profiles, including TG, TC, HDL, and LDL, serve as critical biomarkers of metabolic health, with the LDL/HDL ratio indicating atherogenic risk [41]. In this study, supplementation with LL reduced serum TG content, which was consistent with the previous findings by Siyal et al. [42] LL decreased serum TC level and increased the LDL/HDL ratio, reflecting the enhanced reverse cholesterol transport by LL addition [43]. LL increased LPL, HL, and TL activities of liver in broilers at both day 21 and 50. These findings align with the observation of Ge et al. [24] that bile acids similarly upregulate hepatic LPL in broilers at day 42. Furthermore, Davail et al. [44] showed that decreased LPL activity increases serum TG levels and even leads to excessive fat deposition in the liver of the goose. We hypothesized that dietary LL supplementation could reduce serum TG levels by increasing LPL activity and reducing lipid accumulation in the liver. In addition, the absence of HDL changes despite LDL/HDL ratio improvement, which suggests selective LDL receptor upregulation rather than generalized lipoprotein modulation. These findings collectively indicate that LL supplementation mitigates cardiovascular risk factors while optimizing energy utilization efficiency in broilers, a critical advantage for slow-growing genotypes prone to subcutaneous fat deposition.

Small intestinal morphology serves as a critical indicator of gastrointestinal health and nutrient absorption capacity, such as with VH and VH:CD, which directly correlated with intestinal surface area and absorptive efficiency [45]. Chen et al. [46] showed that LL supplementation increased jejunal VH in white grease-fed broilers receiving LL, aligning with our findings. Brautigan et al. [14] reported that supplementation of LL (1000 g/d) to a combined diet of animal and vegetable fats increased VH and mRNA abundance of collagen genes and collagen staining in jejunal villi. Furthermore, Khonyoung et al. [47] found that LL supplementation promoted the proliferation of intestinal epithelial cells in 49 day old broilers, resulting in increased duodenal mucosal height. In the current experiment, the addition of LL in diets had a positive effect on the VH of both the duodenum and ileum, and VH/CD ratio of the duodenum, jejunum, and ileum throughout the whole period. The beneficial effect of LL on intestinal development may be attributed to the prevention of cell damage and the upregulation of gene expression related to promoting intestinal morphology in broilers [15,48].

## 5. Conclusions

In conclusion, dietary supplementation of LL at 0.05% can improve growth performance, optimize intestinal morphology, enhance lipase activities, and optimize lipid metabolism status in YFBs by regulating the liver lipid metabolism-related enzyme activity. Furthermore, LL supplementation effectively ameliorated the growth-depressive effects in broilers that were fed the low-fat diet.

## Figures and Tables

**Table 1:** Ingredients and nutrient composition of experimental diets during different growth periods.

Items	Day 1–21		Day 22–50	
	3% Lard	2.4% Lard	4.5% Lard	3.6% Lard
Ingredients, %				
Corn, yellow	51.00	51.60	57.03	58.03
Wheat	4.10	4.10	5.00	5.00
Soybean meal	29.00	29.00	21.10	21.00
Cottonseed meal	0.00	0.00	2.00	2.00
Rapeseed meal	0.00	0.00	2.00	2.00
Distillers dried grains with soluble	4.00	4.00	0.00	0.00
Corn gluten meal	4.00	4.00	4.00	4.00
Lard	3.00	2.40	4.50	3.60
Zeolite	0.63	0.63	0.60	0.60
Lime stone	1.50	1.50	1.70	1.70
Calcium hydrogen phosphate	1.40	1.40	0.70	0.70
L-Lysine-HCl	0.07	0.07	0.15	0.15
DL-Methionine	0.12	0.12	0.12	0.12
L-Threonine				
Choline chloride	0.10	0.10	0.05	0.05
Common salt	0.25	0.25	0.25	0.25
Vitamin premix ^1^	0.03	0.03	0.03	0.03
I Mineral premix ^2^	0.30	0.30	0.30	0.30
Antioxidant	0.50	0.50	0.50	0.50
Nutrient composition ^3^				
Crude protein	20.50	20.50	19.50	19.50
Crude protein (analyzed)	20.60	20.49	19.70	19.65
ME (MJ/kg)	12.05	12.01	12.90	12.70
Calcium (analyzed)	0.98	0.99	0.87	0.89
Total phosphorus (analyzed)	0.63	0.61	0.48	0.51
Methionine (analyzed)	0.46	0.47	0.42	0.42
Lysine (analyzed)	1.03	1.04	0.98	0.96

^1^ The vitamin premix provided following per kilogram of diet: vitamin A (transretinyl acetate), 10,000 IU; vitamin D_3_ (cholecalciferol), 3000 IU; vitamin E (all-rac-α-tocopherol acetate), 30 IU; menadione, 1.3 mg; thiamin, 2.2 mg; riboflavin, 8 mg; nicotinamide, 40 mg; choline chloride, 600 mg; calcium pantothenate, 10 mg; pyridoxine·HCl, 4 mg; biotin, 0.04 mg; folic acid, 1 mg; vitamin B_12_ (cobalamin), 0.013 mg. ^2^ The mineral premix provided following per kilogram of diet: Fe (from ferrous sulfate), 80 mg; Cu (from copper sulfate), 8 mg; Mn (from manganese sulfate), 110 mg; Zn (from zinc oxide), 65 mg; I (from calcium iodate), 1.1 mg; and Se (from sodium selenite), 0.3 mg. ^3^ The ME was calculated according to the Tables of Feed Composition and Nutritive Values in China (Xiong et al., 2022) [20].

**Table 2:** The growth performances of 1-to-50-day-old YFBs.

	NF		LF		*p*-Values		
Items	0	+LL	0	+LL	Lard	LL	Lard × LL
1–21 day							
ADG (g/d)	20.20 ± 0.62	20.56 ± 0.29	18.59 ± 0.92	19.40 ± 0.62	<0.001	0.044	0.411
ADFI (g/d)	29.30 ± 0.86	29.42 ± 0.45	28.17 ± 1.11	28.06 ± 1.36	<0.01	0.99	0.795
FCR	1.45 ± 0.01	1.43 ± 0.01	1.52 ± 0.06	1.45 ± 0.03	<0.01	<0.01	0.061
22–50 day							
ADG (g/d)	40.29 ± 2.71	46.90 ± 5.09	38.81 ± 4.02	42.62 ± 4.79	0.087	<0.01	0.391
ADFI (g/d)	86.87 ± 4.96	85.68 ± 8.61	85.76 ± 8.34	86.47 ± 7.20	0.960	0.941	0.766
FCR	2.16 ± 0.08	2.11 ± 0.08	2.21 ± 0.08	2.03 ± 0.07	0.761	<0.01	0.066
1–50 day							
ADG (g/d)	33.28 ± 1.42	35.85 ± 1.92	34.94 ± 1.01	35.83 ± 1.89	0.245	0.021	0.236
ADFI (g/d)	65.65 ± 3.70	66.00 ± 2.94	66.79 ± 1.20	66.71 ± 3.37	0.467	0.913	0.865
FCR	1.99 ± 0.06	1.86 ± 0.04	1.93 ± 0.05	1.88 ± 0.04	0.382	<0.01	0.059

LL = lysolecithin; ADG = average daily gain; ADFI = average daily feed intake; FCR = the feed conversion ratio.

**Table 3:** Nutrient utilization of 19–21-day-old and 47–49-day-old YFBs.

Items	NF		LF		*p*-Value		
	CON	+LL	0	+LL	Lard	LL	Lard × LL
19–21 day							
AME (MJ/kg)	11.66 ± 0.12	11.76 ± 0.20	11.32 ± 0.21	11.60 ± 0.10	<0.01	0.018	0.229
CP, %	61.93 ± 6.18	68.56 ± 9.15	60.19 ± 6.89	67.15 ± 3.52	0.519	0.026	0.980
EE, %	83.26 ± 2.68	87.79 ± 3.32	82.92 ± 3.84	86.86 ± 1.80	0.435	<0.0	0.610
CF, %	39.90 ± 1.98	39.23 ± 1.64	38.58 ± 1.90	39.15 ± 1.37	0.214	0.905	0.529
47–49 day							
AME (MJ/kg)	11.79 ± 0.11	11.95 ± 0.10	11.73 ± 0.16	11.77 ± 0.15	0.045	0.070	0.282
CP, %	64.19 ± 7.25	62.03 ± 3.71	60.46 ± 5.32	63.90 ± 4.75	0.679	0.775	0.221
EE, %	89.58 ± 1.90	88.03 ± 0.94	86.79 ± 2.56	87.51 ± 1.96	0.049	0.607	0.165
CF, %	36.74 ± 2.43	38.84 ± 1.17	37.52 ± 3.00	37.70 ± 1.71	0.845	0.218	0.298

CON = control; AME = apparent metabolizable energy; CP = crude protein; EE = ether extract; CF = crude fiber.

**Table 4:** Lipase activity of 21- and 50-day-old YFBs (U/g).

Items	NF		LF		*p*-Value		
	0	+LL	0	+LL	Lard	LL	Lard × LL
Lipase							
Day 21							
Duodenum	110.94 ± 13.17	141.86 ± 17.55	113.45 ± 16.33	135.38 ± 17.34	0.767	<0.01	0.504
Jejunum	90.76 ± 8.66	106.83 ± 11.43	90.09 ± 8.38	102.99 ± 18.22	0.659	<0.01	0.756
Ileum	45.21 ± 3.71	48.18 ± 6.56	43.53 ± 5.97	48.33 ± 5.95	0.745	0.108	0.697
Pancreas	116.90 ± 24.21	202.00 ± 23.60	114.54 ± 20.50	195.19 ± 20.19	0.666	<0.001	0.834
Day 50							
Duodenum	83.59 ± 14.57	98.77 ± 6.85	80.24 ± 11.17	107.94 ± 9.71	0.522	<0.001	0.176
Jejunum	116.84 ± 24.32	144.50 ± 23.09	113.58 ± 6.47	135.52 ± 14.38	0.428	<0.01	0.709
Ileum	55.63 ± 8.98	62.89 ± 7.64	53.49 ± 6.82	69.47 ± 10.66	0.536	<0.01	0.231
Pancreas	189.82 ± 11.27	258.86 ± 27.35	177.64 ± 78.07	268.13 ± 45.44	0.961	0.019	0.722

**Table 5:** Liver enzymes activity of 21- and 50-day-old broilers (U/g).

Items	NF		LF		*p*-Value		
	0	+LL	0	+LL	Lard	LL	Lard × LL
Day 21							
LPL	4.87 ± 1.18	6.10 ± 1.09	4.67 ± 0.87	6.32 ± 0.40	0.973	<0.01	0.608
HL	2.32 ± 0.42	3.6 ± 0.50	1.72 ± 0.34	3.64 ± 0.47	0.145	<0.001	0.096
TL	7.19 ± 1.36	9.70 ± 1.05	6.39 ± 1.04	9.96 ± 0.76	0.573	<0.001	0.266
Day 50							
LPL	3.15 ± 0.62	3.54 ± 0.46	2.61 ± 0.36	3.72 ± 0.58	0.970	<0.01	0.103
HL	2.17 ± 0.41	2.45 ± 0.22	2.02 ± 0.20	2.59 ± 0.29	0.415	<0.01	0.231
TL	5.31 ± 0.59	5.98 ± 0.48	4.63 ± 0.41	6.31 ± 0.76	0.450	<0.001	0.042

LPL = lipoprotein lipase; HL = hepatic lipase; TL = total lipase.

**Table 6:** Serum lipids indices of 21- and 50-day-old YFBs (mmol/L).

Items	NF		LF		*p*-Value		
	0	+LL	0	+LL	Lard	LL	Lard × LL
Day 21							
Glu	10.52 ± 1.69	16.45 ± 2.45	12.18 ± 2.52	12.09 ± 2.49	0.189	<0.01	<0.011
HDL	2.71 ± 0.27	3.06 ± 0.50	2.82 ± 0.33	2.86 ± 0.26	0.736	0.295	0.196
LDL	1.44 ± 0.15	1.29 ± 0.18	1.41 ± 0.08	1.30 ± 0.18	0.946	0.408	0.133
LDL/HDL	0.48 ± 0.07	0.48 ± 0.07	0.50 ± 0.06	0.46 ± 0.04	0.900	0.522	0.481
TC	4.19 ± 0.51	4.08 ± 0.36	4.34 ± 0.29	4.12 ± 0.44	0.920	0.407	0.455
TG	0.88 ± 0.18	0.54 ± 0.12	0.66 ± 0.17	0.63 ± 0.19	0.376	0.016	0.039
FFA	0.74 ± 0.15	0.26 ± 0.11	0.48 ± 0.16	0.47 ± 0.15	0.668	<0.001	<0.001
Day 50							
Glu	13.79 ± 2.60	13.85 ± 2.51	13.62 ± 2.66	14.98 ± 2.05	0.677	0.537	0.572
HDL	2.20 ± 0.26	2.07 ± 0.35	2.15 ± 0.30	1.77 ± 0.30	0.198	0.06	0.351
LDL	1.01 ± 0.16	1.04 ± 0.19	1.08 ± 0.11	1.06 ± 0.14	0.486	0.989	0.682
LDL/HDL	0.50 ± 0.03	0.46 ± 0.05	0.58 ± 0.13	0.48 ± 0.05	0.119	0.033	0.399
TC	3.50 ± 0.23	2.98 ± 0.57	3.38 ± 0.35	3.01 ± 0.12	0.789	0.013	0.621
TG	0.56 ± 0.19	0.69 ± 0.27	0.73 ± 0.36	0.76 ± 0.22	0.335	0.503	0.687
FFA	0.27 ± 0.13	0.29 ± 0.17	0.30 ± 0.13	0.32 ± 0.13	0.603	0.672	0.989

Glu = glucose; HDL = high-density lipoprotein; LDL = low-density lipoprotein; TC = total cholesterol; TG = triglyceride; FFA = free fatty acids.

**Table 7:** Intestinal histomorphology of 21- and 50-day-old YFBs.

Items	NF		LF		*p*-Value		
	0	+LL	0	+LL	Lard	LL	Lard × LL
Day 21							
Duodenum							
VH	958.26 ± 63.97	1065.21 ± 47.43	917.26 ± 66.70	1018.09 ± 30.26	0.129	<0.01	0.912
CD	195.90 ± 13.07	185.32 ± 9.32	196.08 ± 19.49	201.29 ± 14.20	0.287	0.718	0.297
VH:CD	4.92 ± 0.54	5.75 ± 0.20	4.69 ± 0.17	5.08 ± 0.47	0.036	<0.01	0.266
Jejunum							
VH	953.79 ± 71.15	1072.11 ± 103.77	926.07 ± 69.63	1015.80 ± 135.78	0.412	0.057	0.777
CD	168.76 ± 25.10	139.07 ± 14.17	166.86 ± 17.75	142.38 ± 15.98	0.941	0.013	0.786
VH:CD	5.71 ± 0.53	7.72 ± 0.16	5.58 ± 0.47	7.13 ± 0.37	0.104	<0.001	0.284
Ileum							
VH	830.73 ± 55.95	880.57 ± 34.10	812.73 ± 21.55	872.43 ± 32.63	0.506	0.014	0.800
CD	165.41 ± 11.05	135.95 ± 5.30	163.17 ± 12.72	149.68 ± 10.22	0.282	<0.01	0.143
VH:CD	5.15 ± 0.56	5.95 ± 0.69	5.15 ± 0.37	5.84 ± 0.35	0.834	0.013	0.849
Day 50							
Duodenum							
VH	1482.10 ± 52.51	1630.43 ± 42.28	1513.02 ± 53.97	1644.18 ± 59.94	0.412	<0.001	0.749
CD	205.37 ± 4.29	215.19 ± 7.59	228.59 ± 13.36	215.40 ± 14.68	0.052	0.761	0.055
VH:CD	7.22 ± 0.24	7.40 ± 0.19	6.93 ± 0.23	7.44 ± 0.29	0.322	0.013	0.192
Jejunum							
VH	1495.02 ± 135.50	1687.59 ± 336.50	1490.25 ± 50.91	1647.58 ± 196.78	0.833	0.118	0.868
CD	216.92 ± 13.23	214.75 ± 42.17	221.80 ± 13.17	209.53 ± 37.64	0.804	0.455	0.932
VH:CD	6.89 ± 0.36	7.86 ± 0.65	6.73 ± 0.25	7.45 ± 0.88	0.311	0.012	0.622
Ileum							
VH	1005.42 ± 39.96	1109.04 ± 83.79	996.11 ± 37.27	1086.95 ± 85.95	0.643	0.012	0.850
CD	189.99 ± 20.38	183.17 ± 5.12	194.02 ± 20.65	171.22 ± 12.55	0.630	0.089	0.338
VH:CD	5.28 ± 0.46	6.05 ± 0.42	5.20 ± 0.52	6.10 ± 0.17	0.934	0.002	0.768

VH = villus height; CD = crypt depth.

## Data Availability

The datasets presented in this study are available from the authors on reasonable request.

## References

[1] Sarsenbek A., Wang T., Zhao J.K., Jiang W. (2013). Comparison of carcass yields and meat quality between Baicheng-You chickens and Arbor Acres broilers. Poult. Sci..

[2] Tang H., Gong Y.Z., Wu C.X., Jiang J., Wang Y., Li K. (2009). Variation of meat quality traits among five genotypes of chicken. Poult. Sci..

[3] Ye X., Yu Y., Chen J., Zou Y., Liu S., Tan H., Zhao F., Wang Y. (2022). Evaluation of lipid sources and emulsifier addition on fat digestion of yellow-feathered broilers. J. Anim. Sci..

[4] Fang X., Ye H., Zhang S., Guo L., Xu Y., Zhang D., Nie Q. (2023). Investigation of potential genetic factors for growth traits in yellow-feather broilers using weighted single-step genome-wide association study. Poult. Sci..

[5] Pesti G.M., Bakalli R.I., Qiao M., Sterling K.G. (2002). A comparison of eight grades of fat as broiler feed ingredients. Poult. Sci..

[6] Hossain M.E., Das G.B. (2014). Effect of Crude Soybean Oil Sediment as a Substitute for Refined Soybean Oil in Broiler Diet. Iran. J. Appl. Anim. Sci..

[7] El-Senousey H.K., Wang W., Wang Y., Fan Q., Fouad A.M., Lin X., Gou Z., Li L., Jiang S. (2019). Dietary metabolizable energy responses in yellow-feathered broiler chickens from 29 to 56 d. J. Appl. Poult. Res..

[8] Abdollahi M.R., Ravindran V., Svihus B. (2013). Pelleting of broiler diets: An overview with emphasis on pellet quality and nutritional value. Anim. Feed Sci. Technol..

[9] Maisonnier S., Gomez J., Brée A., Berri C., Baéza E., Carré B. (2003). Effects of microflora status, dietary bile salts and guar gum on lipid digestibility, intestinal bile salts, and histomorphology in broiler chickens. Poult. Sci..

[10] Jansen M., Nuyens F., Buyse J., Leleu S., Van Campenhout L. (2015). Interaction between fat type and lysolecithin supplementation in broiler feeds. Poult. Sci..

[11] Papadopoulos G.A., Poutahidis T., Chalvatzi S., Di Benedetto M., Hardas A., Tsiouris V., Georgopoulou I., Arsenos G., Fortomaris P.D. (2018). Effects of lysolecithin supplementation in low-energy diets on growth performance, nutrient digestibility, viscosity and intestinal morphology of broilers. Br. Poult. Sci..

[12] Liu D., Ma F., Krezhova D. (2011). Soybean phospholipids. Recent Trends for Enhancing the Diversity and Quality of Soybean Products.

[13] Maingret F., Patel A.J., Lesage F., Lazdunski M., Honoré E. (2000). Lysophospholipids open the two-pore domain mechano-gated K+ channels TREK-1 and TRAAK. J. Biol. Chem..

[14] Brautigan D.L., Li R., Kubicka E., Turner S.D., Garcia J.S., Weintraut M.L., Wong E.A. (2017). Lysolecithin as feed additive enhances collagen expression and villus length in the jejunum of broiler chickens. Poult. Sci..

[15] Li X., Shi X., Mesalam N.M., Liu L., Chen Z., Yang B. (2022). Mechanism of Lysoforte in Improving Jejuna Morphology and Health in Broiler Chickens. Front. Vet. Sci..

[16] Zhao P.Y., Kim I.H. (2017). Effect of diets with different energy and lysophospholipids levels on performance, nutrient metabolism, and body composition in broilers. Poult. Sci..

[17] Chen X.D., Ma Q.G., Tang M.Y., Ji C. (2007). Development of breast muscle and meat quality in Arbor Acres broilers, Jingxing 100 crossbred chickens and Beijing fatty chickens. Meat Sci..

[18] Lin X.J., Jiang S.Q., Hong P., Gou Z.Y., Chen F., Li L., Ding F.Y. (2017). Comparison on gastrointestinal digestive enzymes between Ross white chicken and yellow-feather chicken. Chin. Poult..

[19] National Research Council (NRC) (1994). Nutrient Requirements of Poultry.

[20] Xiong B.H., Luo Q.R., Zheng S.S., Zhao Y.G. (2022). Tables of Feed Composition and Nutritive Values in China.

[21] AOAC International (2012). Official Methods of Analysis of AOAC International.

[22] Maiorka A., Dahlke F., Santin E., Kessler A.D.M., Penz A.M. (2004). Effect of energy levels of diets formulated on total or digestible amino acid basis on broiler performance. Braz. J. Poult. Sci..

[23] Niu Z.Y., Shi J.S., Liu F.Z., Wang X., Gao C., Yao L. (2009). Effects of dietary energy and protein on growth performance and carcass quality of broilers during starter phase. Int. J. Poult. Sci..

[24] Ge X.K., Wang A.A., Ying Z.X., Zhang L.G., Su W.P., Cheng K., Feng C.C., Zhou Y.M., Zhang L.L., Wang T. (2019). Effects of diets with different energy and bile acids levels on growth performance and lipid metabolism in broilers. Poult. Sci..

[25] Siyal F.A., Babazadeh D., Wang C., Arain M.A., Saeed M., Ayasan T., Zhang L., Wang T. (2017). Emulsifiers in the poultry industry. Worlds Poult. Sci. J..

[26] Ahmadi-Sefat A.A., Taherpour K., Ghasemi H.A., Gharaei M.A., Shirzadi H., Rostami F. (2022). Effects of an emulsifier blend supplementation on growth performance, nutrient digestibility, intestinal morphology, and muscle fatty acid profile of broiler chickens fed with different levels of energy and protein. Poult. Sci..

[27] Wang Y., Zeng D., Wei L., Chen J., Li H., Wen L., Huang G., Dai Z., Luo J., Sun J. (2024). Effects of emulsifiers on lipid metabolism and performance of yellow-feathered broilers. BMC Vet. Res..

[28] Zhang Z., Zhang S., Nie K., Zheng H., Luo Z., Kim I.H. (2022). Lysolecithin improves broiler growth performance through upregulating growth-related genes and nutrient transporter genes expression independent of experimental diet nutrition level. Animals.

[29] Zaefarian F., Romero L.F., Ravindran V. (2015). Influence of high dose of phytase and an emulsifier on performance, apparent metabolisable energy and nitrogen retention in broilers fed on diets containing soy oil or tallow. Br. Poult. Sci..

[30] Allahyari-bake S., Jahanian R. (2016). Effects of Dietary Fat Source and Supplemental Lysophosphatidylcholine on Performance, Immune Responses, and Ileal Nutrient Digestibility in Broilers Fed Corn/soybean Meal-or Corn/wheat/soybean Meal-based Diets. Poult. Sci..

[31] Zampiga M., Meluzzi A., Sirri F. (2016). Effect of dietary supplementation of lysophospholipids on productive performance, nu-trient digestibility and carcass quality traits of broiler chickens. Ital. J. Anim. Sci..

[32] Wealleans A.L., Jansen M., Di Benedetto M. (2020). The addition of lysolecithin to broiler diets improves growth performance across fat levels and sources: A meta-analysis of 33 trials. Br. Poult. Sci..

[33] Wiseman J., Salvador F. (1991). The influence of free fatty acid content and degree of saturation on the apparent me-tabolizable energy value of fats fed to broilers. Poult. Sci..

[34] Zhang B., Haitao L., Zhao D., Guo Y., Barri A. (2011). Effect of fat type and lysophosphatidylcholine addition to broiler diets on performance, apparent digestibility of fatty acids, and apparent metabolizable energy content. Anim. Feed. Sci. Technol..

[35] Tancharoenrat P., Ravindran V., Zaefarian F., Ravindran G. (2014). Digestion of fat and fatty acids along the gastroin-testinal tract of broiler chickens. Poult. Sci..

[36] Tenório K.I., Eyng C., Nunes R.V., Duarte C.R.D.A., Souza C.D., Junior N.R., Köhler T.L., Tesser G.L.S. (2025). Emulsifier in broiler diets containing deactived full-fat soybean. Rev. Bras. Zootec..

[37] Tavares F.B., de Souza Lima K.R., Manno M.C., Barata Y.M.L., de Oliveira Pinheiro H.C., Arruda J.D.C.B., Faturi C. (2022). Effect of exogenous emulsifier and different fat sources on the performance, carcass characteristics, nutrient digestibility, and serum lipid profile of broiler chickens. Braz. J. Vet. Res. Anim. Sci..

[38] Lai W., Huang W., Dong B., Cao A., Zhang W., Li J., Wu H., Zhang L. (2018). Effects of dietary supplemental bile acids on performance, carcass characteristics, serum lipid metabolites and intestinal enzyme activities of broiler chickens. Poult. Sci..

[39] Merkel M., Weinstock P.H., Chajek-Shaul T., Radner H., Yin B.Y., Breslow J.L., Goldberg I.J. (1998). Lipoprotein lipase expression exclusively in liver. A mouse model for metabolism in the neonatal period and during cachexia. J. Clin. Investig..

[40] Mersmann H.J. (1998). Lipoprotein and hormone-sensitive lipases in porcine adipose tissue. J. Anim. Sci..

[41] Helkin A., Stein J.J., Lin S., Siddiqui S., Maier K.G., Gahtan V. (2016). Dyslipidemia Part 1—Review of Lipid Metabolism and Vascular Cell Physiology. Vasc. Endovasc. Surg..

[42] Siyal F.A., El-Hack M.E.A., Alagawany M., Wang C., Wan X.L., He J.T., Wang M.F., Zhang L.L., Zhong X., Wang T. (2017). Effect of soy lecithin on growth performance, nutrient digestibility and hepatic antioxidant parameters of broiler chickens. Int. J. Pharmacol..

[43] Mooradian A.D., Haas M.J. (2014). The effect of nutritional supplements on serum high-density lipoprotein cholesterol and apolipoprotein AI. Am. J. Cardiovasc. Drugs.

[44] Davail S., Guy G., Andre J.M., Hermier D., Hoo-Paris R. (2000). Metabolism in two breeds of geese with moderate or large overfeeding induced liver-steatosis. Comp. Biochem. Physiol. A Mol. Integr. Physiol..

[45] Hosoyamada Y., Sakai T. (2007). Mechanical components of rat intestinal villi as revealed by ultrastructural analysis with special reference to the axial smooth muscle cells in the villi. Arch. Histol. Cytol..

[46] Chen C., Jung B., Kim W.K. (2019). Effects of lysophospholipid on growth performance, carcass yield, intestinal development, and bone quality in broilers. Poult. Sci..

[47] Khonyoung D., Yamauchi K., Suzuki K. (2015). Influence of dietary fat sources and lysolecithin on growth performance, visceral organ size, and histological intestinal alteration in broiler chickens. Livest. Sci..

[48] Skoura A., Hla T. (2009). Lysophospholipid receptors in vertebrate development, physiology, and pathology. J. Lipid Res..

